# A Sprayable Nanoplatform Breaks the Vicious Cycle of Diabetic Wounds via Photoactivated Antioxidant and Drug Delivery

**DOI:** 10.1002/advs.75886

**Published:** 2026-05-29

**Authors:** Jiahao Guo, Yuanyuan Meng, Qixiang Gui, Xi‐Tao Yang, Yong Fan, Xiaodong Zhu

**Affiliations:** ^1^ Department of Nanomedicine Naval Medical University Shanghai P. R. China; ^2^ Department of Chemistry State Key Laboratory of Molecular Engineering of Polymers Shanghai Key Laboratory of Molecular Catalysis and Innovative Materials and Ichem Fudan University Shanghai P. R. China; ^3^ School of Medicine Department of Plastic and Reconstructive Surgery of Shanghai East Hospital Tongji University Shanghai P. R. China; ^4^ Department of Interventional Therapy Multidisciplinary Team of Vascular Anomalies Shanghai Ninth People's Hospital Shanghai Jiaotong University School of Medicine Shanghai P. R. China

**Keywords:** cascade reaction, diabetic wound healing, MXene, nanozymes, synergistic therapy

## Abstract

Diabetic wound healing is still a challenge, because the pathological microenvironment is characterized by a vicious cycle of oxidative stress, hypoxia, infection and chronic inflammation. Herein, we report a near‐infrared (NIR) light‐activated cascade nanodressing incorporated into a sprayable hyaluronic acid hydrogel. Under NIR irradiation, MXene serves not only as a photothermal substrate but also generates an interfacial electronic effect that drastically enhances the multienzyme‐mimicking activities of zinc hexacyanoferrate (ZnHCF), enabling efficient ROS clearance and alleviation of hypoxia. The mild photothermal effect further triggers‐controlled release of deferoxamine (DFO), which cooperates with ZnHCF to chelate free iron ions, thereby suppressing ROS generation at the source. The system also exhibits synergistic photothermal‐nanoknife‐Zn^2+^ antibacterial efficacy, promoting macrophage repolarization from an M1 to an M2 phenotype. Moreover, DFO‐induced HIF‐1α upregulation facilitates angiogenesis and collagen deposition. Both in vitro and in vivo studies have confirmed that this sprayable nanodressing with “3A” (anti‐inflammatory, antibacterial, and angiogenic) effect can significantly accelerate the healing of diabetic wounds by improving the chronic wound microenvironment, achieving complete re‐epithelization within 13 days. This work sets the stage for a new generation of on‐demand, activated, synergistic systems, offering a viable path toward treating refractory tissue injuries.

## Graphical abstract

A sprayable near‐infrared‐activated nanoplatform incorporating MXene, ZnHCF nanozyme, and deferoxamine efficiently breaks the vicious cycle of diabetic wounds. Upon irradiation, interfacial electron transfer and photothermal effects enhance multi‐enzyme activity, enabling explosive ROS elimination, alleviation of hypoxia, and controlled DFO release. This synergistic 3A strategy achieves accelerated, complete wound healing.

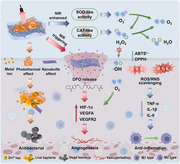

## 1 Introduction

The global diabetes population has reached a staggering 537 million and is projected to grow by 46% to 784 million by 2045, with 19%–34% of these patients facing amputation risk due to chronic wounds such as diabetic foot ulcers [1, 2, 3]. The difficulty in healing these wounds stems from an imbalance in inflammatory repair. This vicious cycle is rooted in its special pathological microenvironment, which is characterized by the continuous overproduction of reactive oxygen species/nitrogen species (ROS/RNS) and chronic hypoxia [4, 5, 6]. This strong oxidative stress state will cause direct biomolecular damage, make the polarization of pro‐inflammatory M1 macrophages permanent, and intensify the inflammatory reaction, thus creating a harmful microenvironment [7, 8]. Furthermore, inhibition of angiogenesis will also seriously delay the healing process, thus making the pathological microenvironment permanent [3, 9]. A hyperglycemic microenvironment in diabetic wounds can promote the degradation of hypoxia‐inducible factor‐1α (HIF‐1α), inhibit the expression of vascular endothelial growth factor (VEGF), and thus hinder angiogenesis. Moreover, diabetic wounds are highly susceptible to bacterial infection, aggravating local hypoxia and inflammation, resulting in a vicious cycle of infection and tissue necrosis [10, 11, 12]. Therefore, the existing treatment strategies are often ineffective because they cannot synergistically disintegrate this interrelated pathological network [13]. There is an urgent need for a collaborative and innovative program to control infection, eliminate inflammation, and promote angiogenesis, so as to break this vicious circle [14, 15].

Under physiological conditions, redox homeostasis is maintained by endogenous enzyme defense systems, such as superoxide dismutase (SOD) and catalase (CAT), which can jointly remove excess ROS [16]. However, in the diabetic wound microenvironment, mitochondrial dysfunction and abnormal activation of NADPH oxidase induced by continuous hyperglycemia lead to the production of a large number of ROS/RNS, which exceed the endogenous antioxidant capacity [17]. To eliminate this imbalance, antioxidant biomaterials, especially two‐dimensional MXenes (such as Ti_3_C_2_), have attracted great interest [18, 19]. It not only has significant photothermal conversion efficiency and inherent antioxidant activity, but also has antibacterial properties, good biocompatibility, and rich surface functional groups [20, 21]. Despite these advantages, the practical application of pristine MXene is limited by its weak enzyme catalytic efficiency and critical oxidative degradation sensitivity [22]. This self‐sacrificing behavior quickly damages its photothermal and antioxidant properties, thus limiting the effectiveness of long‐term treatment.

To solve these limitations, Modifying MXenes to enhance the performance of powerful nanozymes can be a feasible strategy [23, 24, 25]. Hexacyanoferrates (HCFs) are a kind of Prussian blue analogues with adjustable metal centers, high biological safety, and inherent multi‐enzyme‐like activities [26, 27, 28]. It is worth noting that HCF‐based nanomaterials such as magnesium and calcium ferricyanide have shown effective ROS clearance and anti‐inflammatory abilities in various disease models, highlighting the extensive potential of this material family in regulating the pathological microenvironment [29, 30]. In this case, zinc hexacyanoferrate (ZnHCF) is an attractive candidate material. The incorporation of Zn^2+^ ions provides inherent antibacterial and anti‐inflammatory properties, while the hexacyanoferrate framework confers potent SOD/CAT‐like activities, enabling the sequential elimination of ROS and alleviation of hypoxia [31, 32]. The integration of this multifunctional nanozyme and MXene can also form a protective heterostructure, thereby enhancing antioxidant stability. Moreover, under NIR radiation, the potential of the interface electron effect to enhance the catalytic activity of MXene‐nanozyme heterojunctions provides a promising way for a photoactivation system [33, 34, 35]. While addressing the initial oxidative and infectious injury represents only the first step, the ultimate goal of functional tissue regeneration lies in actively promoting angiogenesis to drive the subsequent healing stage. In this regard, iron chelator deferoxamine (DFO), as a clinically approved medicine, can not only inhibit the iron‐catalyzed Fenton reaction to supplement antioxidant defense but also directly regulate the strong pro‐angiogenic response by stabilizing HIF‐1α [36]. As a result, the integration of DFO into a responsive nanoplatform can bridge the gap between antioxidant therapy and successful tissue regeneration.

**SCHEME 1:**
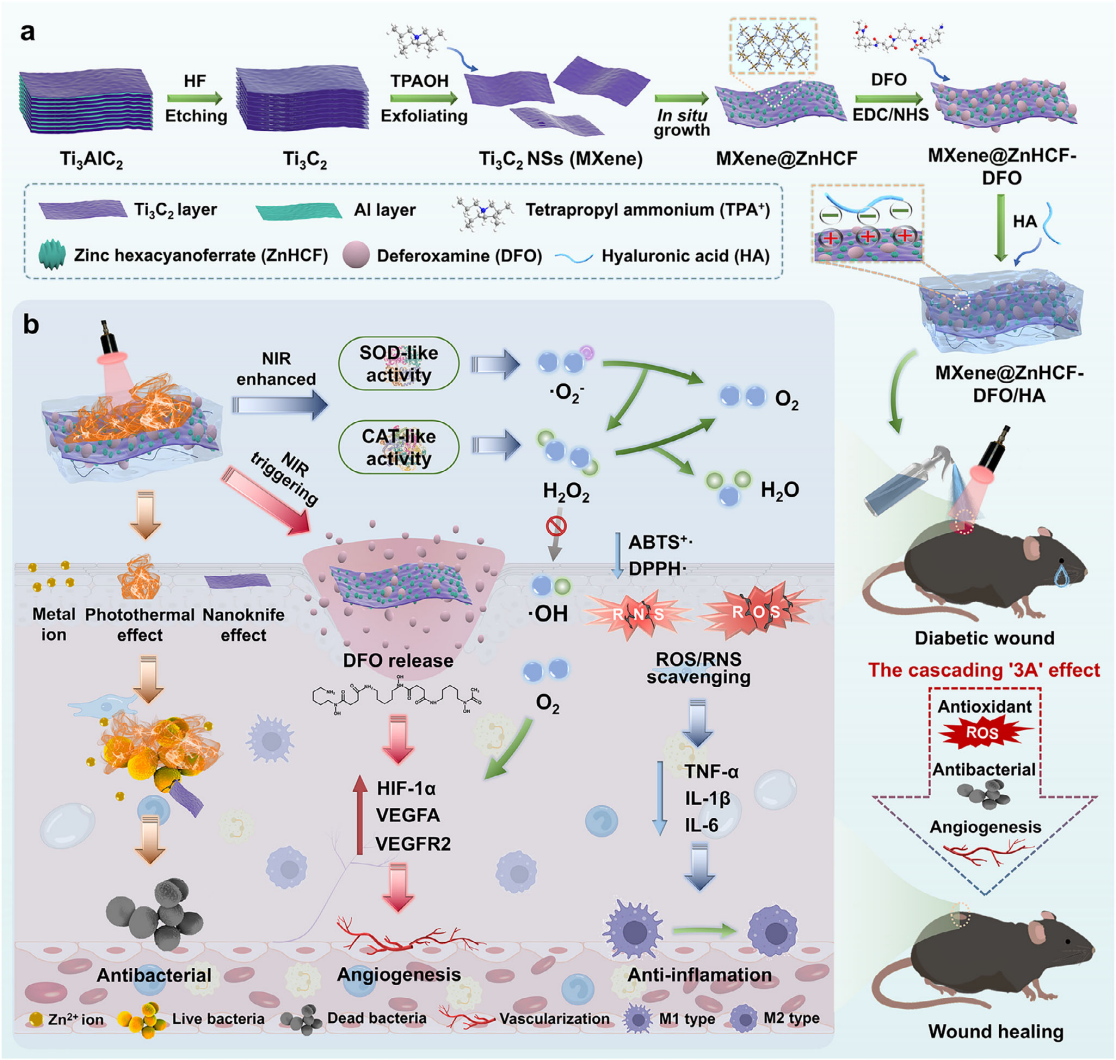
Schematic diagram of spray preparation and light‐activated repair mechanism of chronic diabetic wounds. (a) The preparation of nanoplatform spray involves the synthesis of layered d‐Ti_3_C_2_, the construction of photothermal‐catalytic MXene@ZnHCF, the loading of DFO, and the encapsulation of HA. (b) The NIR‐activated nanoplatform exhibits the “3A” (anti‐inflammatory, antibacterial, and angiogenic) effect, rapidly repairing diabetic wounds.

Herein, we propose a “3A” (antioxidant, antibacterial, and angiogenic) strategy to break the vicious cycle of diabetic wounds using a NIR‐activated cascade nanoplatform, MXene@ZnHCF‐DFO/HA, integrated into a sprayable hyaluronic acid hydrogel for facile, conformal application to irregular wound surfaces (Scheme 1a). The design leverages MXene's excellent photothermal conversion efficiency and interface electron effect to significantly enhance the multi‐enzyme simulation activity of ZnHCF under NIR radiation (Scheme 1b), thereby eliminating ROS and improving on‐demand hypoxia. The mild photothermal treatment further triggered the controlled release of DFO, which interacted with ZnHCF to chelate free iron ions, thus inhibiting the Fenton reaction and enhancing antioxidant defense. Moreover, due to the inherent Zn^2+^‐mediated antibacterial activity and the physical bactericidal effect based on photothermal/MXene, the platform has played a powerful synergistic antibacterial effect. These multiple effects, including efficient ROS clearance, relief from hypoxia, strong antibacterial properties, and the promotion of macrophage polarization from an M1 phenotype to an M2 phenotype, have cooperatively reshaped the harsh wound microenvironment. Reprogramming of wound microenvironment can further enhance the stability and angiogenesis of HIF‐1α driven by DFO, which is conducive to tissue regeneration. In vivo and in vitro experiments have shown that this sprayable smart nano dressing can significantly accelerate the healing of diabetic wounds by creating a microenvironment conducive to regeneration and “3A” effect.

## 2 Results and Discussion

### 2.1 Preparation and Characterization

**FIGURE 1:**
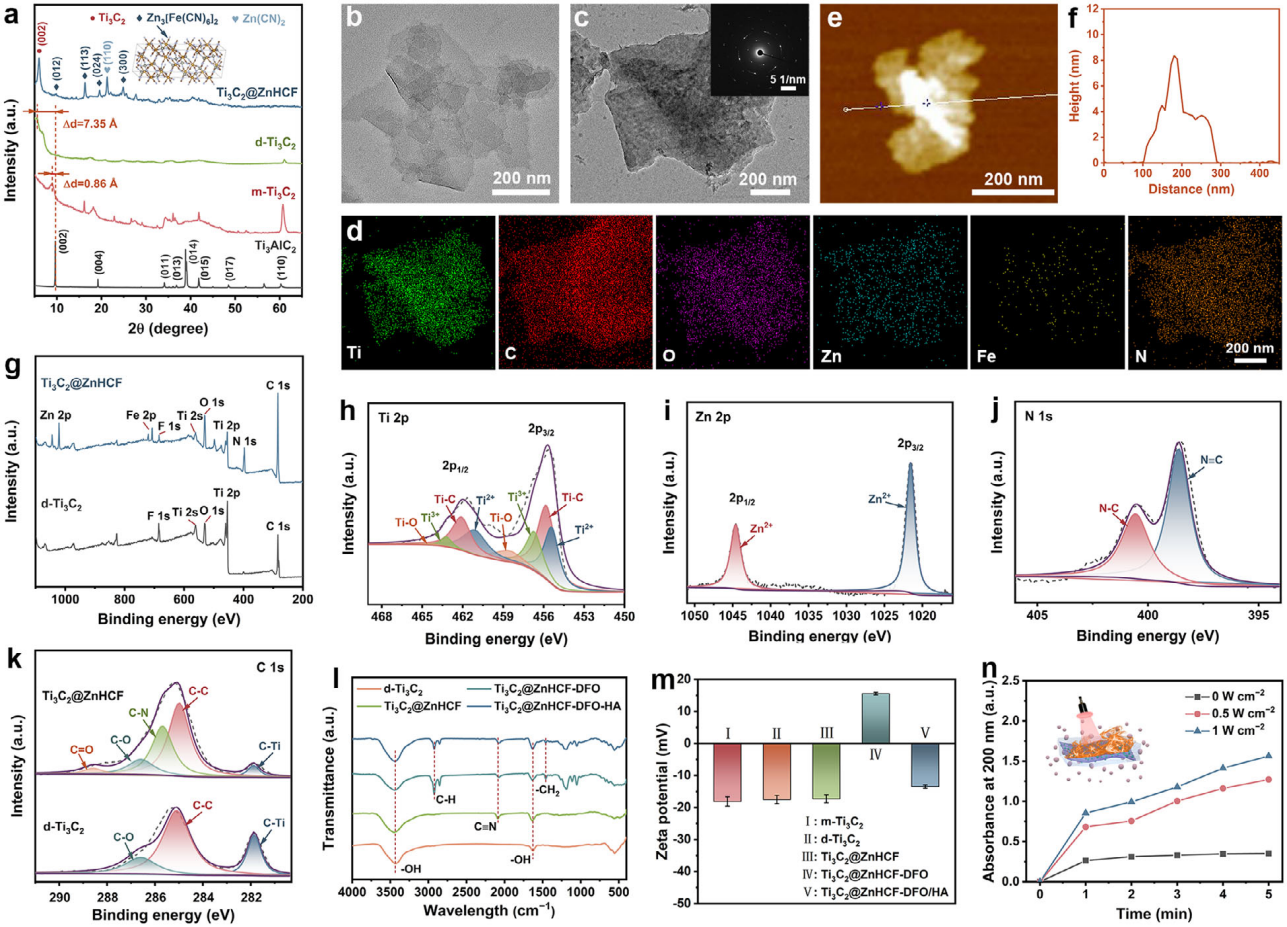
Preparation and characterization of cascade antioxidant and drug‐delivery nanoplatform. (a) XRD patterns of Ti_3_AlC_2_, m‐Ti_3_C_2_, d‐Ti_3_C_2_, and d‐Ti_3_C_2_@ZnHCF. (b) A TEM image of d‐Ti_3_C_2_ nanosheets. (c) A TEM image of MXene@ZnHCF (the insets: the corresponding SAED profile) and (d) corresponding element mappings. (e) An AFM image of MXene@ZnHCF and (f) corresponding profile height. (g) XPS spectra of d‐Ti_3_C_2_ and MXene@ZnHCF. High‐resolution (h) Ti 2p, (i) Zn 2p, and (j) N 1s XPS spectra of MXene@ZnHCF. (k) High‐resolution C 1s XPS spectra of d‐Ti_3_C_2_ and MXene@ZnHCF. (l) FTIR spectroscopy of d‐Ti_3_C_2_, MXene@ZnHCF, MXene@ZnHCF‐DFO, and MXene@ZnHCF‐DFO/HA. (m) Zeta potential analysis of m‐Ti_3_C_2_, d‐Ti_3_C_2_, MXene@ZnHCF, MXene@ZnHCF‐DFO, and MXene@ZnHCF‐DFO/HA (*n* = 3). (n) Release curve of light‐controlled DFO.

The synthesis of the MXene@ZnHCF heterojunctions commenced with the preparation of Ti_3_C_2_ MXene nanosheets from a Ti_3_AlC_2_ MAX phase precursor. This was achieved through a two‐step process involving HF etching to obtain a multi‐layered accordion‐like structure (Figure S1), followed by delamination to yield single‐layer nanosheets (Figure 1b). The success of this exfoliation was confirmed by x‐ray diffraction (XRD), where the characteristic peaks (PDF‐#52‐0875) of the Ti_3_AlC_2_ precursor diminished, and the (002) peak shifted to a lower angle, signifying an increased interlayer spacing and successful purification (Figure 1a). Subsequently, the affinity of MXene surface pores for metal cations was utilized to generate zinc hexacyanoferrate (ZnHCF) nanoparticles in situ on its surface (Figure 1c), and corresponding elemental mappings showed that the characteristic elements of Ti, C, O, Zn, Fe, and N were evenly distributed on the nanosheets (Figure 1d) [37]. The XRD diffraction peaks further confirm that the ZnHCF loaded on MXene was mainly in the crystal forms of Zn_3_[Fe(CN)_6_]_2_ (PDF‐#38‐0688) and Zn(CN)_2_ (PDF‐#06‐0175). The successful loading of ZnHCF was also corroborated by atomic force microscopy (AFM), which showed an increase in the thickness of the nanosheets from ∼2 nm for pristine MXene (Figure S2) to ∼4‐8 nm for the MXene@ZnHCF heterojunctions (Figure 1e,f). Further research on the surface composition and chemical states of MXene@ZnHCF was conducted through x‐ray photoelectron spectroscopy (XPS) analysis (Figure 1g). As shown in Figure 1h, the high‐resolution Ti 2p spectrum of MXene@ZnHCF was deconvoluted into eight peaks, attributed to Ti─O (464.6 and 458.7 eV), Ti^3+^ (463.1 and 456.7 eV), Ti─C (462.1 and 455.8 eV), and Ti^2+^ (461.1 and 455.4 eV) [38, 39]. Furthermore, the presence of ZnHCF was verified by the appearance of characteristic peaks for Zn 2p (Zn^2+^ at 1044.5 and 1021.5 eV) and N 1s, the latter resolved into contributions from N─C (400.5 eV) and N≡C (398.6 eV) bonds (Figure 1i,j) [40, 41]. To assess the stability of heterostructures in physiological‐like environments, MXene@ZnHCF was cultured in PBS for 3 days. XPS analysis (Figure S3) showed that MXene was slightly oxidized, with increased Ti─O bonding. However, the composition of ZnHCF remained stable, and the Zn 2p binding energy did not change significantly, ensuring continuous catalytic and antibacterial activity. As shown in Figure 1k, compared to those of MXene, the C 1s peaks of MXene@ZnHCF can additionally fit C═O (288.5 eV) and C─N (285.7 eV), which were absent in pristine MXene, providing further evidence for the successful coordination of the ZnHCF structure on the MXene surface [42].

The as‐synthesized MXene@ZnHCF were subsequently functionalized with deferoxamine (DFO) via a Schiff base reaction, followed by encapsulation in hyaluronic acid (HA) via electrostatic self‐assembly. HA‐based microgels are biocompatible and biodegradable polymers that have been utilized in drug delivery and biomedical engineering [43, 44]. This final step yielded the sprayable microgel formulation, designated MXene@ZnHCF‐DFO/HA (MZDH), for diabetic wound management. According to the Fourier transform infrared (FTIR) spectroscopy (Figure 1l), the characteristic peaks at 1632 and 3429 cm^−1^ are the bending and stretching vibration of ─OH, and the characteristic peak at 2091 cm^−1^ is the stretching vibration of C≡N [45]. Critically, new peaks emerged at 2919 and 1459 cm^−^^1^, corresponding to C─H and ‐CH_2_ stretching vibrations, respectively, which are attributable to the alkyl chains of the grafted DFO molecules [46]. Further evidence for the successful assembly of the nanoplatform was provided by dynamic light scattering and zeta potential analyses. The hydrodynamic diameter of MZDH microgel is about 548 nm, and it is monodisperse (PDI ∼0.3, Figure S4). A notable shift in surface potential from negative (−17.3 mV) to positive (15.5 mV) after DFO grafting (Figure 1m) facilitated the subsequent electrostatic adsorption of the anionic HA polymer. Collectively, these physicochemical characterizations confirm the successful fabrication of the MZDH microgels.

### 2.2 Photothermal‐Enhanced Cascaded Oxidation Resistance

**FIGURE 2:**
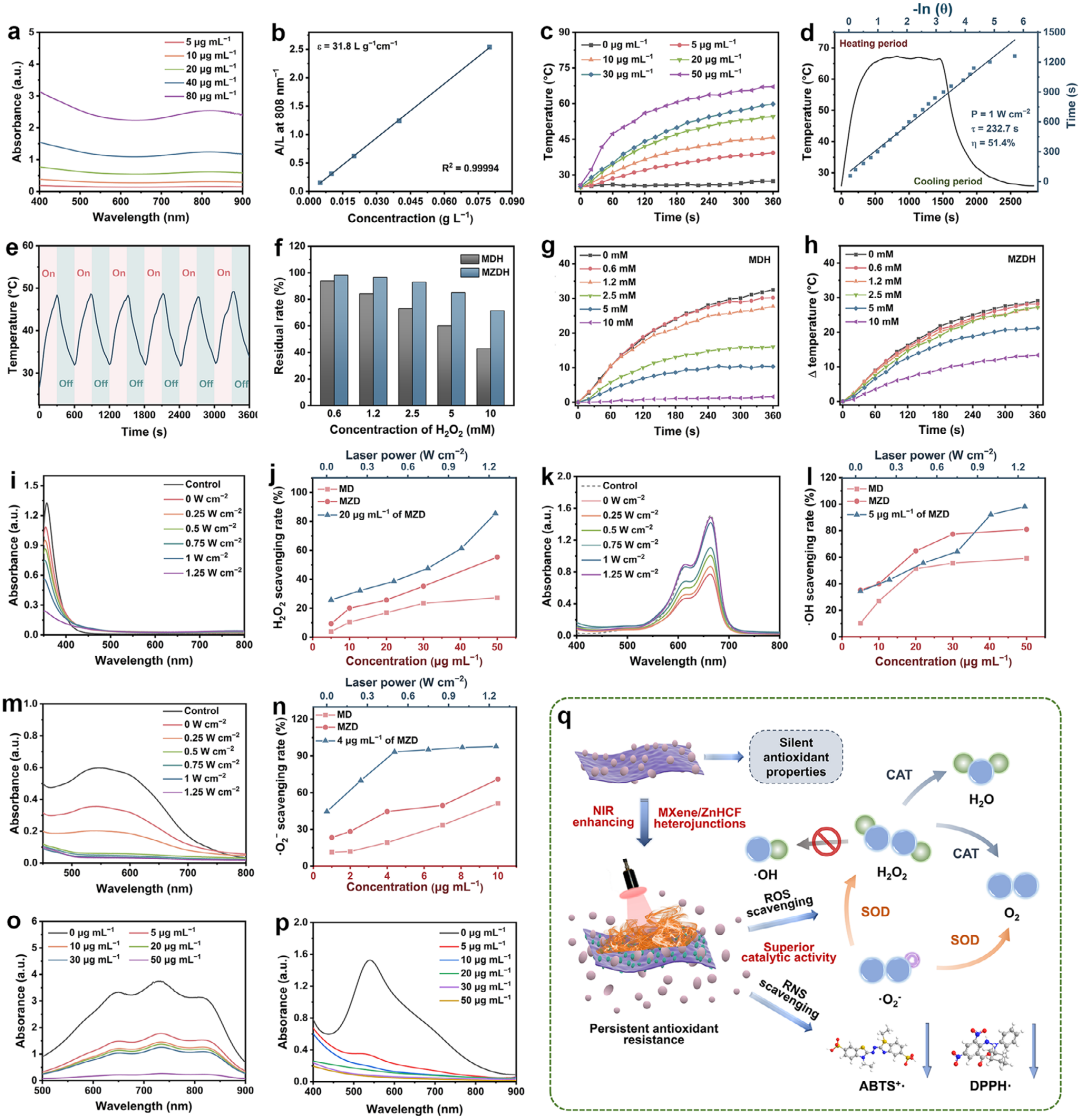
Photothermal‐enhanced cascaded oxidation resistance of nanoplatforms. (a) Absorption spectra of MZDH, and (b) the corresponding extinction coefficient (ε) fitted at 808 nm. (c) Temperature change curve of MZDH with different concentrations under 808 nm irradiation. (d) The complete temperature rise/fall curve of MZDH under laser irradiation and the corresponding photothermal conversion efficiency (η). (e) The temperature variation curve of MZDH over six on/off cycles under 808 nm irradiation. (f) After incubation with MDH and MZDH in different concentrations of H_2_O_2_, the residual sample rate was obtained by calculating the absorbance. Temperature rise curves of (g) MDH and (h) MZDH in different concentrations of H_2_O_2_ under NIR irradiation. (i) UV–vis absorbance of 20 µg mL^−1^ MZD in the CAT‐mimicking process at different laser powers. (j) H_2_O_2_ scavenging rate of different concentrations of MD and MZD, and 20 µg mL^−1^ MZD under different laser powers. (k) UV–vis absorbance of 5 µg mL^−1^ MZD in •OH scavenging process at different laser power. (l) •OH scavenging rate of different concentrations of MD and MZD, and 5 µg mL^−1^ MZD under different laser powers. (m) UV–vis absorbance of 4 µg mL^−1^ MZD in the SOD‐mimicking process at different laser powers. (n) •O_2_^−^ scavenging rate of different concentrations of MD and MZD, and 5 µg mL^−1^ MZD. UV–vis absorbance of MZD with different concentrations for (o) ABTS^+^• and (p) DPPH• scavenging process. (q) Schematic diagram of NIR and heterostructure‐enhanced cascade antioxidant and drug release of highly stable nanoplatforms.

To validate the on‐demand drug release capability, the DFO release profile from MZDH was investigated under near‐infrared (NIR) laser irradiation. As shown in Figure 1n, NIR irradiation triggered a significant and rapid increase in DFO release. The MZDH microgels exhibited a concentration‐dependent absorption profile in the NIR region (Figure 2a). Based on the Lambert–Beer law, the extinction coefficient (ε) of MZDH at 808 nm was determined to be 31.8 L g^−1^ cm^−1^ (Figure 2b), indicating its strong potential as an efficient photothermal agent [24]. The real‐time temperature changes of MZDH excited by NIR were recorded by a micro infrared thermal imaging platform to analyze the photothermal effect (Figure S5). Correspondingly, under 808 nm irradiation, the MZDH dispersions with concentrations of 20 and 50 µg mL^−1^ reached temperatures of 54.5°C and 67.1°C within 6 min, respectively. In contrast, the PBS control showed negligible heating (Figure 2c). When the laser power increased to 1.5 W cm^−2^, the temperature of 20 µg mL^−1^ MZDH reached a high value of 72.3°C at 6 min (Figure S6). After a complete heating and natural cooling analysis, the photothermal conversion efficiency (η) of MZDH was calculated to be 51.4% (Figure 2d). Additionally, MZDH also exhibits excellent photothermal stability in six on/off laser cycles (Figure 2e). Given the susceptibility of MXene to oxidation in physiological environments, which can lead to self‐sacrificial degradation and performance decay, the stability of MXene‐DFO/HA (MDH), and MZDH in oxidants was investigated (Figure S7) [47]. As shown in Figure 2f, MZDH showed significantly higher residual mass (71.4% with 10 mm H_2_O_2_) than that of MDH (42.8% with 10 mm H_2_O_2_) after treatment with different concentrations of H_2_O_2_, indicating that ZnHCF coating can effectively protect the MXene substrate from oxidation. Subsequently, further simulations were conducted to investigate the changes in photothermal performance in a strongly oxidizing environment (Figure 2g,h). MZDH has excellent oxidation resistance, particularly when the H_2_O_2_ concentration is below 2.5 mm; its photothermal performance is hardly affected (Figure S8).

Then, the scavenging efficiency for various reactive oxygen/nitrogen species (ROS/RNS) related to wound healing damage was systematically evaluated. The catalase (CAT)‐like activity, which decomposes H_2_O_2_ into H_2_O and O_2_ to alleviate oxidative damage and hypoxia, was assessed via the ammonium molybdate colorimetric method [48]. As shown in Figure S9, both MD and MZD induced a concentration‐dependent decrease in absorbance at 360 nm, indicating H_2_O_2_ consumption. Notably, MZD exhibited a more pronounced H_2_O_2_ elimination under 808 nm irradiation, suggesting that the heterostructure effectively enhances CAT‐like activity in a photo‐responsive manner (Figures 2i,j). To quantitatively evaluate the catalytic efficiency, kinetic parameters of the CAT‐like activity were determined using the Lineweaver‐Burk method. As summarized in Figure S10, the MZD exhibited a lower K_M_ value than MD, indicating greater substrate affinity. Under NIR irradiation, the Vmax was substantially increased, demonstrating that the combination of interfacial electronic effects and mild photothermal heating synergistically boosts the enzymatic turnover. SOD‐mimicking nanozymes can eliminate various free radicals that are continuously generated during normal body metabolism, such as catalyzing the dismutation reaction of •O_2_^−^ to O_2_ and H_2_O_2_, thereby improving oxidative stress in the wound microenvironment [49]. Firstly, H_2_O_2_ catalyzes the •OH formation of the most oxidizing endogenous free radical through an iron‐dependent Fenton reaction [50]. After co‐incubation with the sample, the MB indicator is added to detect the residual amount of •OH. As illustrated in Figure S11, the heterostructure effectively eliminated •OH, an effect attributed to the synergistic iron chelation by the HCF framework and DFO, which suppressed •OH generation [30, 36]. 808 nm irradiation significantly enhanced •OH removal, with near‐complete scavenging observed at higher power densities (Figures 2k,l). The •O_2_^−^ scavenging ability was analyzed by measuring the inhibitory effect on the reduction of nitroblue tetrazolium (NBT), and the absorbance at 560 nm decreased with the increase in the amount of heterostructure (Figure S12). Under 808 nm irradiation, the heterojunction achieved a remarkable •O_2_^−^ clearance rate of 93.3% (Figure 2m,n).

RNS are nitrogen‐containing radicals generated by the reaction of NO with active substances in the body, usually induced by an inflammatory reaction [51]. The scavenging capacity toward RNS was assessed using ABTS^+^• and DPPH• as stable nitrogen‐centered radical models. A decrease in absorbance at 730 nm (ABTS^+^•) and 530 nm (DPPH•) indicated efficient elimination (Figures S13a and S14a). The heterostructure substantially removed both ABTS^+^• and DPPH• radicals (Figure 2o,p), with the MXene@ZnHCF composition markedly enhancing RNS clearance (Figures S13b and S14b). Similarly, as shown in the newly added Figure S15, the Nyquist plots reveal that the MXene@ZnHCF heterojunction exhibits a significantly smaller semicircle diameter than pristine ZnHCF, indicating a marked decrease in charge‐transfer resistance. This observation demonstrates that incorporating MXene greatly facilitates interfacial charge transfer, likely through the formation of a Schottky‐like heterojunction that promotes electron migration from MXene to ZnHCF upon NIR excitation. In summary, this highly stable cascade nanoplatform can significantly enhance multi‐enzyme‐mimicking activity through interfacial electron transfer from MXene to ZnHCF, promoting the fluidity of reactants under an NIR‐activated photothermal effect (Figure 2q). This significantly enhances the scavenging ability against various reactive oxygen species and alleviates hypoxia, while inhibiting the formation of the highly reactive •OH.

### 2.3 Assessment of the Biocompatibility and Promoting Effect on Cell Migration

**FIGURE 3:**
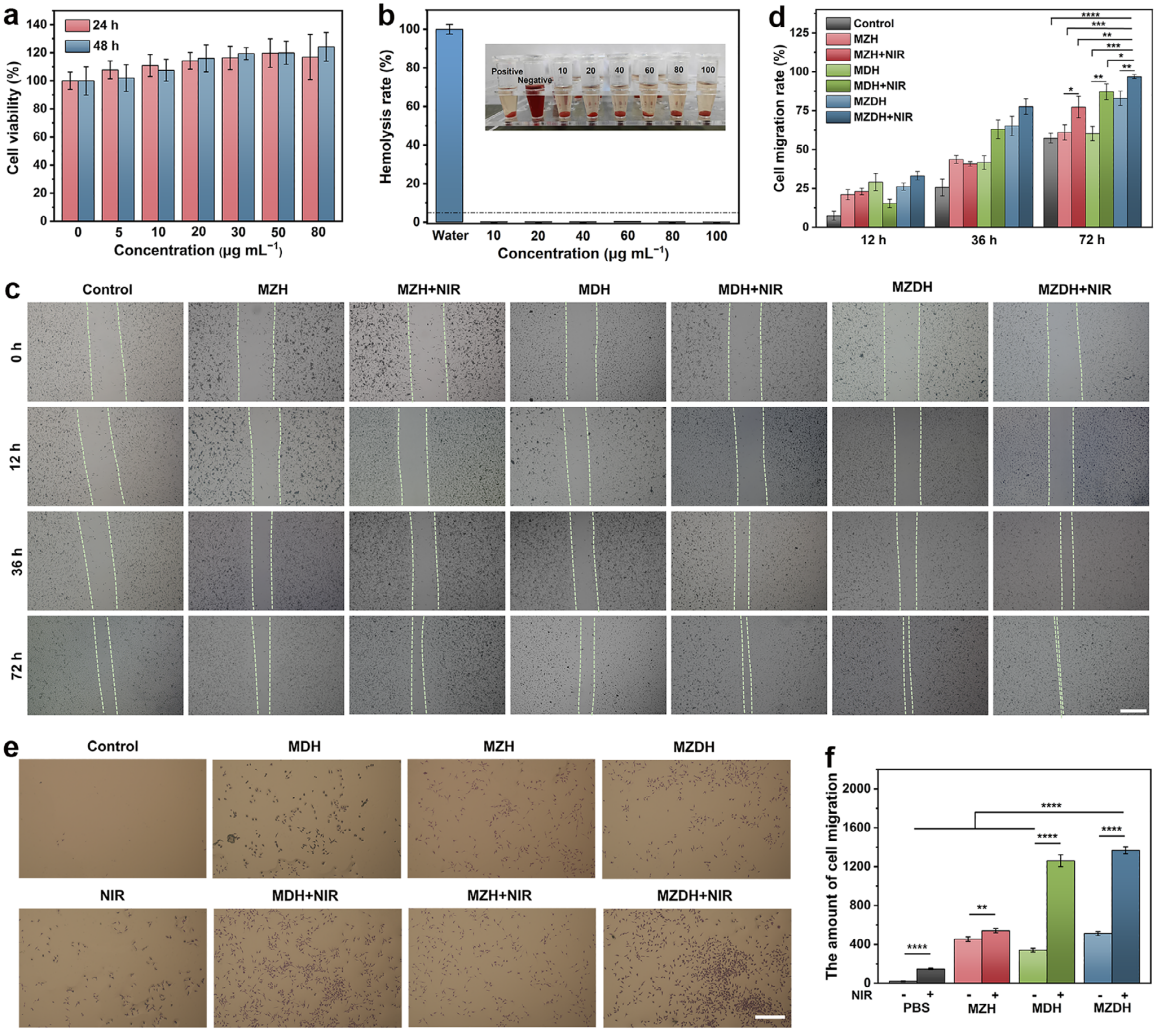
Assessment of the biocompatibility of cascade‐antioxidant and drug‐delivery nanoplatforms and their promoting effect on cell migration. (a) Relative cell viability of L929 cells after incubation with MZDH dispersion of different concentrations for 24 and 48 h (*n* = 5). (b) Quantification of hemolysis after incubation of MZDH and red blood cells at different concentrations (*n* = 5). (c) Scratch assay after co‐incubation of L929 cells and MZDH for different durations (scale bar = 100 µm) and (d) corresponding quantification analysis (*n* = 3). (e) Crystal violet staining image of cells in the lower chamber of the transwell in the cell migration experiment (scale bar = 200 µm) and (f) corresponding quantification analysis (*n* = 3). Statistical significance is assessed by unpaired Student's two‐sided *t*‐test, and asterisks indicate significant differences (^*^ *p* < 0.05, ^**^ *p* < 0.01, ^***^ *p* < 0.001, and ^****^ *p* < 0.0001).

To evaluate the biosafety of the developed microgels for diabetic wound therapy, their cytotoxicity was assessed by co‐incubating L929 fibroblasts with various concentrations of MZDH. Using the standard CCK‐8 assay, MZDH with different concentrations exhibited great biocompatibility at both 24 and 48 h, and even exhibits a promoting effect on cell proliferation (Figure 3a). When MZDH is co‐incubated with cells and irradiated with lasers of different powers, it can also promote cell proliferation (Figure S16). Especially at 0.75 and 1 W cm^−2^, the proliferation effect is most significant, due to the cell‐proliferation‐promoting effect of mild photothermal therapy (mPTT) [52, 53]. Furthermore, the excellent hemocompatibility of MZDH was confirmed by a hemolysis assay. After co‐incubation with red blood cells, MZDH induced negligible hemolysis, well below the safe threshold of 5% (Figure 3b), indicating its high blood compatibility.

To further evaluate the role of MZDH in promoting cell proliferation and migration, which is the key link in the process of wound healing. The scratch test of L929 fibroblasts showed that MZDH could significantly promote cell migration within 72 h, which may be related to the reduction of oxidative stress by MZDH (Figure 3c). The NIR radiation further enhances this migration effect (Figure 3d). These results were confirmed by the supplemented Transwell migration experiment, which showed that the mild photothermal effect of MZDH significantly stimulated the directional migration of cells to the inferior chamber (Figures 3e,f). In conclusion, these results confirm that MZDH has good biological activity. In particular, upon NIR activation, it can significantly promote cell proliferation and migration, demonstrating its strong potential as an advanced wound dressing. To investigate the cellular targets of MZDH, FITC‐labeled nanoparticles were co‐incubated in L929 fibroblasts and RAW264.7 macrophages (Figure S17). Fluorescence imaging revealed rapid and sustained uptake by L929 cells within 2–8 h, whereas RAW264.7 cells showed minimal association until 8 h. This differential uptake pattern suggests that MZDH primarily acts on tissue‐resident cells, with its anti‐inflammatory effects likely mediated by microenvironmental modulation rather than by direct internalization of immune cells.

### 2.4 The Antibacterial Ability

**FIGURE 4:**
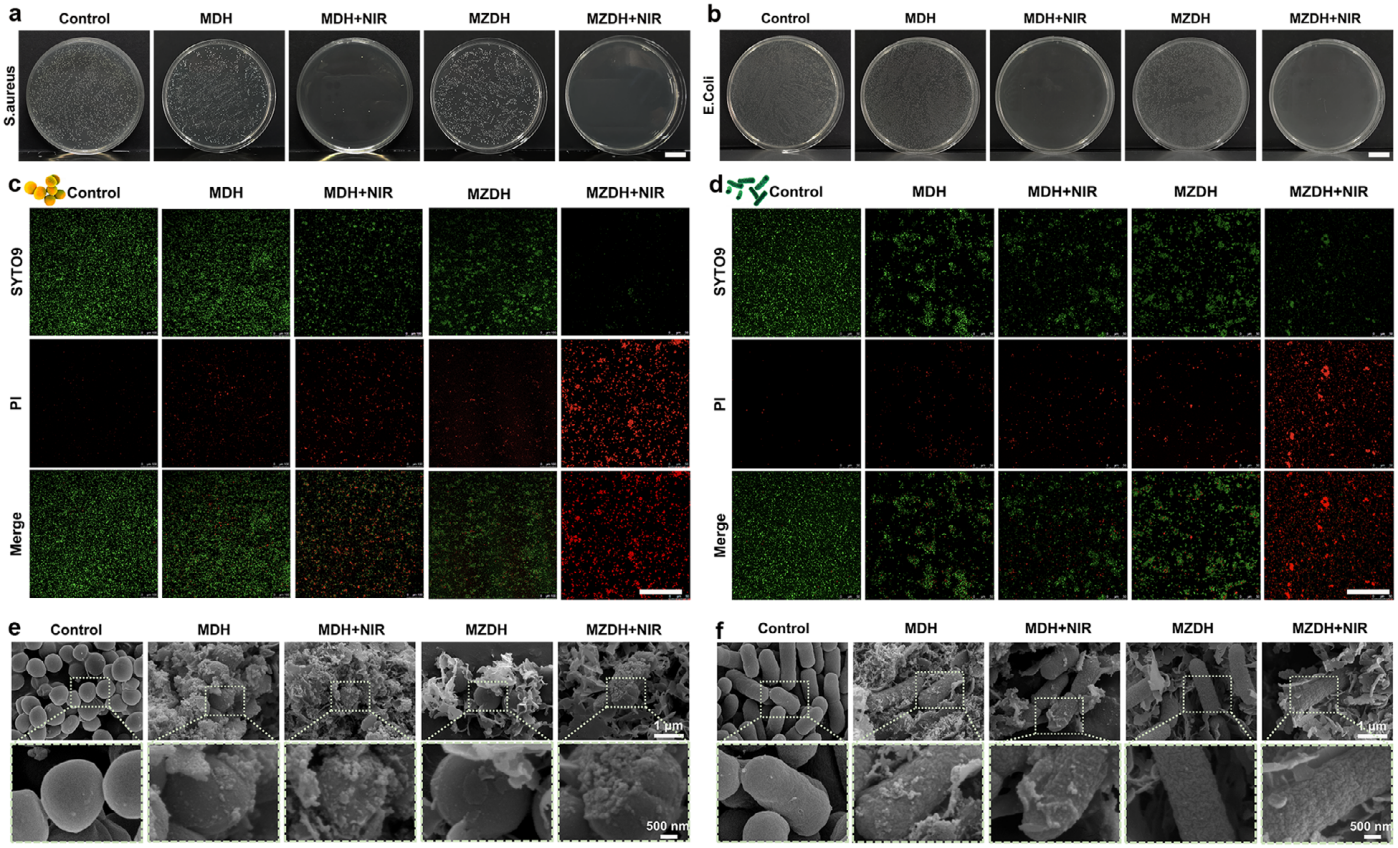
The antibacterial ability of nanoplatforms. Coating plate count of different drugs against (a) *S. aureus* and (b) *E. coli* (scale bar = 2 cm). Use SYTO9 and PI to perform live/dead staining on (c) *S. aureus* and (d) *E. coli* subjected to different treatments, and observe them using CLSM (scale bar = 100 µm). SEM images of (e) *S. aureus* and (f) *E. coli* after different treatments.

Given the high susceptibility of diabetic wounds to bacterial infection, the antibacterial efficacy of the MZDH microgel was evaluated against typical Gram‐positive (Staphylococcus aureus, *S.aureus*) and Gram‐negative (Escherichia coli, *E.coli*) bacteria. As illustrated in Figure 4a,b, both MDH and MZDH treatments reduced bacterial colony formation compared to the control. This antibacterial effect was markedly enhanced under NIR irradiation, with the MZDH+NIR group exhibiting no visible colonies and achieving antibacterial rates exceeding 99% against both strains (Figure S18). This excellent performance is attributed to the synergistic mechanism of photothermal ablation and Zn^2+^ mediated bactericidal activity. Moreover, the photothermal effect will also promote the dissociation of HA in the outer layer of the microgel, exposing the positively‐charged MZD nanosheets, which are easy to adhere to the bacterial membrane, and their sharp edges can lead to the physical damage of bacteria and the leakage of contents [54, 55]. A confocal laser scanning microscope (CLSM) was utilized to further confirm the vitality of bacteria by live/dead staining, showing that the number of live bacteria decreased sharply after MZDH+NIR treatment (Figure 4c,d), which was consistent with the plate counting results. Observing the morphology of *S.aureus* (Figure 4e) and *E.coli* (Figure 4f) subjected to different treatments through SEM, the cell membrane of the control group was intact and smooth, exhibiting complete spherical and rod‐like shapes, respectively. After the addition of different sample treatments, the surface morphology of the bacteria was affected to varying degrees, and a large number of nanosheets were attached. In the MZDH+NIR group, *S.aureus* seemed to exhibit content leakage, while the surface of *E.coli* became more wrinkled. When the bacterial cell membrane is damaged, it will not be able to maintain its inherent morphology, control the transport of metabolites, protect against external damage, and perform many other physiological functions, leading to a profound loss of bacterial viability. In conclusion, the NIR activation of the MZDH nanoplatform can achieve efficient bacterial eradication synergy through various mechanisms.

### 2.5 In Vitro Anti‐Inflammatory and Pro‐Angiogenic Effects and Mechanisms

**FIGURE 5:**
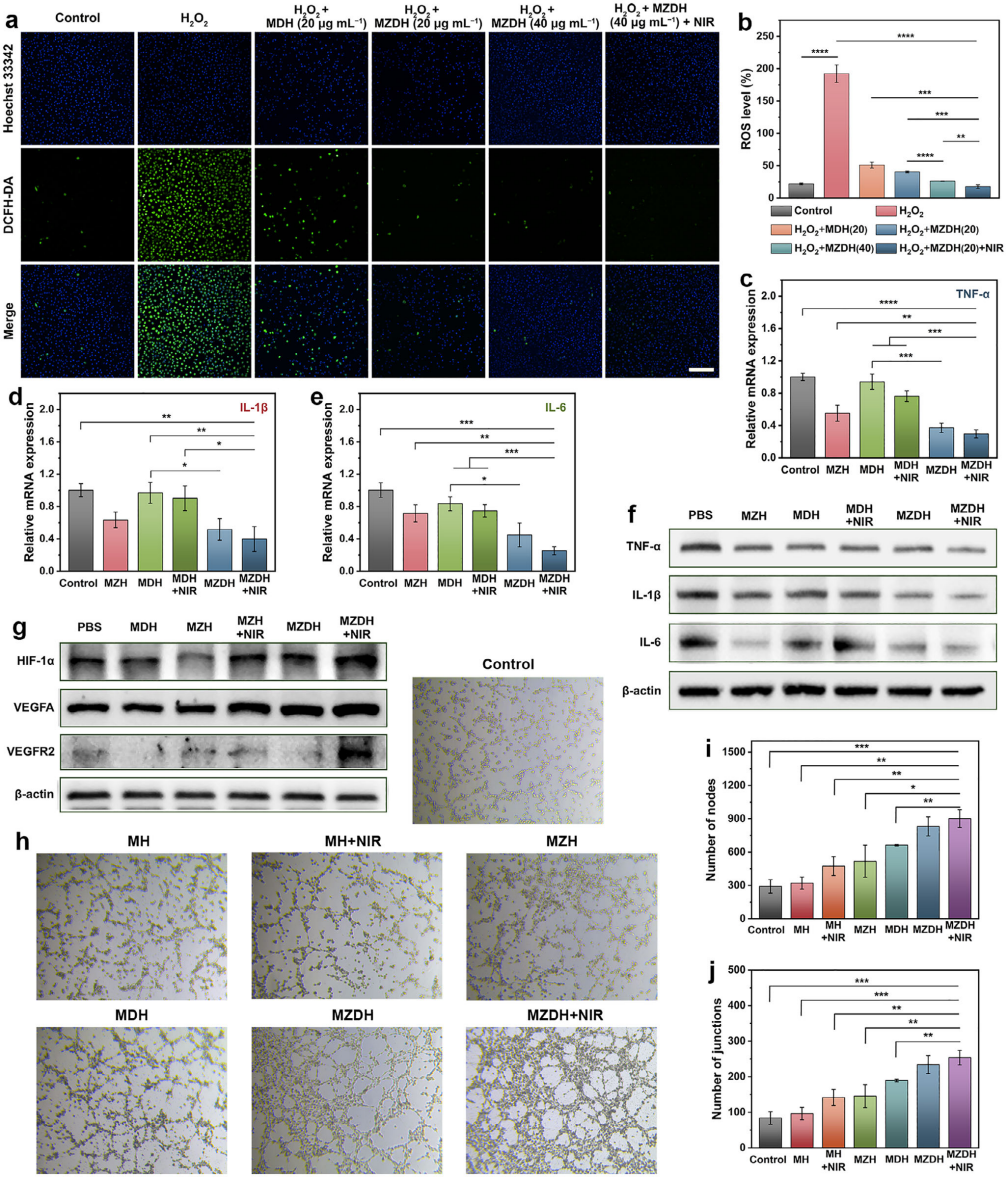
In vitro anti‐inflammatory and pro‐angiogenic effects and mechanisms of nanoplatforms. (a) CLSM images of L929 cells with ROS after different treatments (scale bar: 200 µm, blue fluorescence indicates the cell nucleus, and green fluorescence indicates ROS) and (b) corresponding quantification of cellular ROS level (*n* = 3). The detection of the mRNA levels of (c) TNF‐α, (d) IL‐1β, and (e) IL‐6 after different treatments using qRT‐PCR (*n* = 3). (f) The detection of the expression of inflammation‐related proteins using Western blot. (g) The detection of the expression of vascular‐related protein using Western blot. (h) In vitro angiogenesis experiment after different treatments and corresponding quantification of (i) nodes and (j) junctions (*n* = 3). Statistical significance is assessed by unpaired Student's two‐sided t‐test and asterisks indicate significant differences (^*^ *p* < 0.05, ^**^ *p* < 0.01, ^***^ *p* < 0.001, and ^****^ *p* < 0.0001).

The impaired healing of diabetic wounds is largely driven by a self‐perpetuating cycle of inflammation‐repair imbalance, fueled by persistent oxidative stress and dysregulated immune responses in the wound microenvironment [56]. To assess whether the cascade antioxidant nanoplatform can protect cells from such oxidative damage, we first established an H_2_O_2_‐induced oxidative stress model in L929 fibroblasts (Figure 5a). The H_2_O_2_ group showed a high ROS level, while MDH, MZDH, and high‐concentration MZDH could effectively eliminate excess intracellular ROS (Figure 5b). It is noteworthy that under near‐infrared radiation, MZDH even reduces the level of ROS to below that of the non‐stressed control, indicating its potent and light‐enhanced antioxidant capacity.

Further evaluation of the immunomodulatory effect of nanoplatforms on macrophage polarization is crucial for transitioning from the wound inflammation stage to the proliferation stage. Lipopolysaccharide (LPS)‐stimulated macrophages were used as an in vitro inflammatory model, and the expression of typical M1 macrophage markers (TNF‐α, IL‐1β, and IL‐6) was analyzed by RT‐qPCR. As shown in Figure 5c–e, MXene@ZnHCF (MZ) heterojunction could significantly inhibit these pro‐inflammatory cytokines, and the expression level of MZDH+NIR group was the lowest, which was 0.45 (TNF‐α), 0.35 (IL‐1β), and 0.33 times (IL‐6) lower than that of the control group, respectively. Further analysis of three representative inflammation‐related factors using Western Blot (WB) yielded nearly identical results to those obtained from qPCR (Figure 5f and Figure S19). These results indicate that the MZDH nanoplatform can effectively reduce oxidative stress and promote the resolution of inflammation.

A pivotal factor impeding diabetic wound healing is the failure of neovascularization, largely attributable to hyperglycemia‐induced degradation of hypoxia‐inducible factor‐1α (HIF‐1α), which in turn suppresses the expression of vascular endothelial growth factor (VEGF)‐ a central regulator that enhances vascular permeability and promotes endothelial cell proliferation [57]. Therefore, the effect of drug‐delivery nanoplatforms on the protein expression of HIF‐α, VEGFA, and VEGFR2 promoting angiogenesis in L929 cells was detected and analyzed by WB (Figure 5g) [58]. The small molecule drug DFO, an FDA‐approved hypoxia mimetic, can inhibit prolyl hydroxylase and induce HIF‐1α, and upregulate the expression of VEGF and other key angiogenesis‐related factors [36]. Therefore, to distinguish between oxygenation improvement and DFO‐driven HIF‐1α stabilization, comparative analyses across control groups with varying functionalities were performed. When drug‐delivery nanoplatforms are activated by NIR to release DFO, the expression of HIF‐1α, VEGFA, and VEGFR2 proteins significantly increases (Figure S20), indicating that the nanoplatform activates the endogenous angiogenesis pathway. The functional impact of the nanoplatform on vascular morphogenesis was further assessed using a tube formation assay with human umbilical vein endothelial cells (HUVECs) (Figure 5h). The results show that the MZDH+NIR group has the most significant promoting effect on HUVEC cell angiogenesis, with the number of nodes (Figure 5i) and junctions (Figure 5j) reaching 900.7 ± 79.8 and 253.7 ± 20.5, respectively. These results collectively demonstrate that the cascade‐antioxidant and drug‐delivery nanoplatform not only mitigates oxidative stress and inflammation but also potently stimulates angiogenesis, highlighting its multifaceted “3A” antioxidant, antibacterial, and angiogenic) therapeutic potential to promote diabetic wound regeneration.

### 2.6 Evaluation of Promoting Wound Healing in Diabetic Mice

**FIGURE 6:**
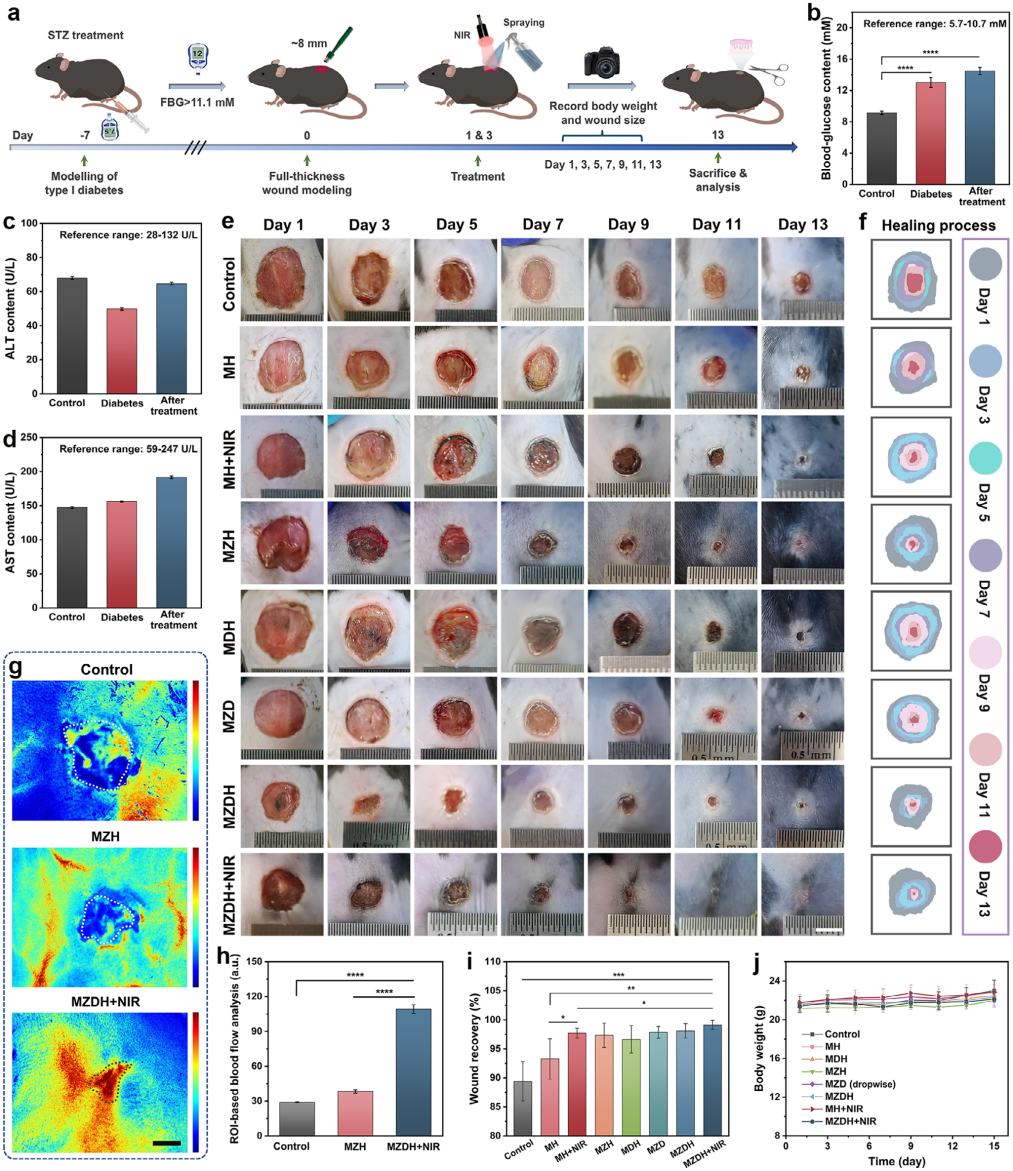
Evaluation of cascade‐antioxidant and drug‐delivery nanoplatforms on promoting wound healing in diabetic mice. (a) Schematic diagram of the modeling and treatment process for full‐skin damage in diabetic mice. Blood biochemical analysis of diabetic mice before and after treatment, including (b) blood glucose, (c) ALT, and (d) AST (*n* = 5). (e) Digital photos of the healing process of skin wounds in diabetic mice during the treatment cycle, with the first treatment on Day 1, and (f) corresponding wound healing trajectory diagram. (g) Blood flow imaging map of the wound site in diabetic mice on day 13 (scale bar = 1 mm) and (h) corresponding ROI‐based blood flow analysis (*n* = 5). (i) Quantitative analysis of the final wound healing rate (*n* = 5). (j) Changes in body weight of mice during different treatment processes (*n* = 5). Statistical significance is assessed by unpaired Student's two‐sided *t*‐test, and asterisks indicate significant differences (^*^ *p* < 0.05, ^**^ *p* < 0.01, ^***^ *p* < 0.001, and ^****^ *p* < 0.0001).

Based on in vitro mechanistic exploration, a model of full‐thickness skin injury (Φ = 8 mm) in type I diabetes was established in C57 mice to evaluate the therapeutic potential of the cascade‐antioxidant and drug‐delivery nanoplatforms (Figure 6a). The diabetic mice were randomly assigned to 8 experimental groups: control, MH, MH+NIR, MZH, MDH, MZD, MZDH, and MZDH+NIR. Prior to assessing the therapeutic mechanisms, systemic biocompatibility was verified through routine blood tests (Figure S21) and blood biochemical analysis in normal, diabetic, and post‐treatment mice (Figure 6b–d and Figure S22). Blood glucose measurements confirmed the successful establishment of the diabetic model (>10.7 mm), while all hematological and biochemical parameters remained within normal ranges across all groups, indicating the favorable biosafety profile of the developed nanoplatforms.

To evaluate the repair effect of the nanoplatform on diabetic wounds, the weight and wound healing of mice were monitored every other day during the 13‐day treatment period. Representative wound images and quantitative analysis showed healing trends in all groups (Figure 6e,f). The MZDH+NIR group showed healing as early as the fifth day, featured by no exudate and a significantly reduced wound size. On day 9, all wounds began to scab, and the wound healing rate in the MZDH+NIR group reached 91.1%, while the other wounds had relatively larger areas (Figure S23). On the 13th day, the wound healing rate of MZDH+NIR treatment was 99.1% (Figure 6i), almost completely re‐epithelialized, while the eschar in other groups continued to exist. Blood flow perfusion within the wound bed, monitored via laser speckle imaging (Figure 6g), demonstrated enhanced angiogenesis in the MZDH+NIR group, with significantly higher perfusion compared to the control and MZH groups (Figure 6h). Throughout the study, all mice maintained stable body weights (Figure 6j), indicating no systemic adverse effects. On the 28th day after MZDH+NIR treatment, histological analysis of the main organs revealed no abnormalities (Figure S24), further confirming the nanoplatform's long‐term safety. As a result, the NIR‐activated cascade‐antioxidant and drug‐delivery nanoplatform effectively promotes wound healing in diabetic mice by mitigating wound exudation, enhancing local blood perfusion, and accelerating tissue repair.

### 2.7 Transcriptome Sequencing Analysis of Molecular Mechanism of Wound Repair

**FIGURE 7:**
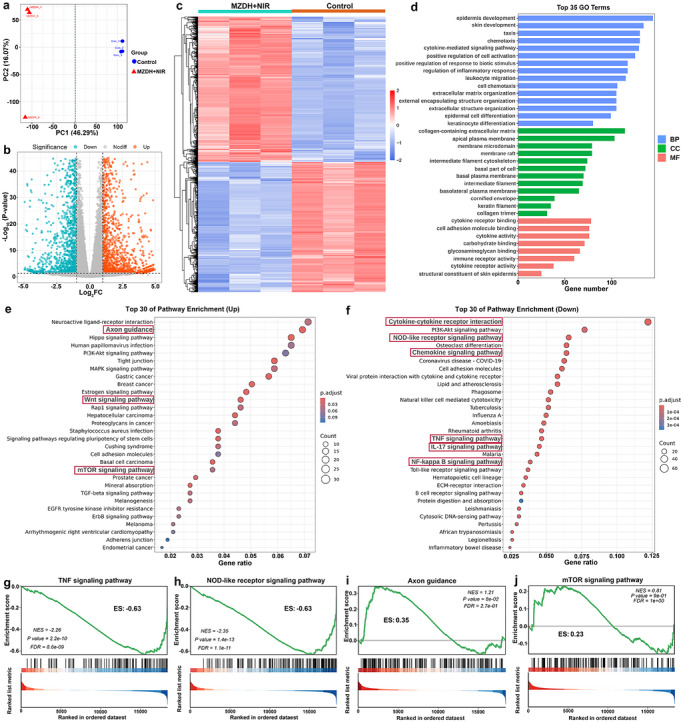
Analyze the molecular mechanisms underlying wound repair using transcriptomic sequencing. (a) PCA analysis of the control and MZDH+NIR groups. (b) The difference gene volcano map and (c) heat map between the control group and MZDH+NIR group (p < 0.05, flod change ≥ 2). (d) Significant enrichment of GO items for genes in two groups (Biological process: top 15; Cellular components: top 12; Molecular function: top 8). KEGG enrichment analysis revealed (e) upregulated genes (top 30) and (f) downregulated genes (top 30) (Pathways related to wound repair are highlighted in red.). The GSEA plots of the (g) TNF signaling pathway, (h) NOD‐like receptor signaling pathway, (i) axon guidance, and (j) mTOR signaling pathway obtained from regulated gene pathways using the KEGG database.

After confirming that NIR‐activated MZDH microgel promotes significant healing of diabetic wounds, the molecular mechanism underlying its regulation of inflammation and tissue repair was further clarified through transcriptome analysis. As shown in Figure 7a and Figure S25, principal component analysis (PCA) revealed significant clustering between the control and treatment groups, indicating substantial differences in the overall gene expression profile. The results of the comparative analysis showed that 3232 genes were differentially expressed, including 1704 up‐regulated and 1528 down‐regulated genes (Figure 7b). The corresponding thermogram further confirmed the significant biological effect of NIR‐activated MDH and MZDH treatment on the wound healing process (Figure 7c and Figure S26). As shown in Figure 7d and Figure S27, through gene ontology (GO) enrichment analysis of DEGs, differential mechanisms involving immune receptor activity, regulation of inflammatory response, cytokine receptor binding, positive regulation of cell activation, collagen‐containing extracellular matrix, and epidermis/skin development were identified. Compared with the Control group, the MDH+NIR group showed only modest enrichment in immune‐ and inflammation‐related pathways, whereas the MZDH+NIR group exhibited robust regulation of these biological processes. Notably, GO terms associated with muscle tissue development, contraction, and cytoskeletal organization were also differentially regulated, suggesting that MZDH+NIR can regulate wound healing by coordinating immune responses, extracellular matrix remodeling, and epithelial cell development pathways.

Further analysis of the signaling mechanism of MZDH+NIR treatment effect through the Kyoto Encyclopedia of genes and genomes (KEGG) pathway. This indicates that gene expression in several key pathways related to wound healing is significantly upregulated, including the Wnt, mTOR, and axon guidance pathways (Figure 7e). The Wnt signaling pathway is involved in key processes of cell proliferation, differentiation, and tissue pattern formation. mTOR is an atypical serine/threonine kinase that plays a central role in the regulation of metabolism, autophagy, and cell growth, which are necessary for tissue regeneration [59, 60]. On the contrary, multiple immune‐related pathways were significantly downregulated (Figure 7f). Relative to MZDH+NIR, MDH+NIR displayed weaker modulation of inflammatory pathways (Figure S28). Conversely, MZDH+NIR downregulated these pro‐inflammatory cascades more effectively, consistent with its superior anti‐inflammatory and pro‐angiogenic effects observed in vivo. Moreover, gene set enrichment analysis (GSEA) further corroborated these findings, showing significant positive and negative enrichment of gene sets related to these biological processes (Figure 7g–j and Figure S29). In conclusion, transcriptomic profiling demonstrates that MZDH+NIR promotes diabetic wound healing by coordinating immune responses, enhancing cellular activation, and stimulating tissue‐forming programs.

### 2.8 Histological Evaluation of the Wound Healing Process

**FIGURE 8:**
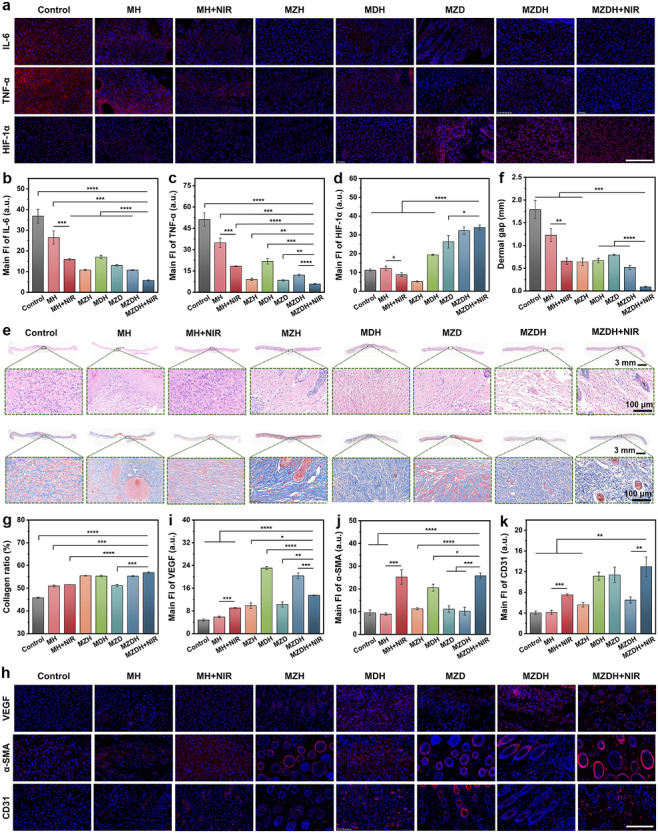
Histological evaluation of the wound healing process. (a) Immunofluorescence staining section images of wound tissues on day 7 in different treatment groups (scale bar: 200 µm) and corresponding quantitative analysis of (b) IL‐6, (c) TNF‐α, and (d) HIF‐1α (*n* = 3). (f) H&E and Masson staining images of wound tissues on day 13 in different treatment groups and corresponding quantitative analysis of (e) dermal gap and (g) collagen ratio (*n* = 3). (h) Immunofluorescence staining section images of wound tissues on day 13 in different treatment groups (scale bar: 200 µm) and corresponding quantitative analysis of (i) VEGF, (j) α‐SMA, and (k) CD31 (*n* = 3). Statistical significance is assessed by unpaired Student's two‐sided t‐test and asterisks indicate significant differences (^*^ *p* < 0.05, ^**^ *p* < 0.01, ^***^ *p* < 0.001, and ^****^ *p* < 0.0001).

To elucidate the mechanisms by which the cascade‐antioxidant and drug‐delivery nanoplatform promotes wound healing, comprehensive histopathological and immunofluorescence analyses on wound sections to evaluate epidermal regeneration, inflammatory regulation, collagen deposition, and angiogenesis were conducted. Hematoxylin and eosin (H&E) and Masson's trichrome staining of day‐7 wound tissues (inflammatory/proliferative phase) showed that the wound healing of the MZDH+NIR group was the most obvious (Figure S30), which was consistent with the wound healing trajectory, and the collagen deposition degree of each group was different. The transition from M1 to M2 macrophages is essential for reducing inflammation and promoting a normal proliferative state. Staining for pro‐inflammatory markers of IL‐6 and TNF‐α (Figure 8a) revealed a significant decrease in expression in the group containing MZ heterojunctions, with the MZDH+NIR group exhibiting the most pronounced anti‐inflammatory effect (Figure 8b,c). Meanwhile, immunofluorescence detected that HIF‐1α (the key regulator of angiogenesis factor) was highly expressed in the group containing the DFO component, especially under the NIR activation (Figure 8d). These results collectively indicate that the nanoplatform accelerates progression into the proliferation/remodeling phase by regulating immune polarization, enhancing collagen tissue, and stimulating angiogenic signals.

To further clarify the tissue regeneration‐promoting ability of the nano platform, the wound sections were analyzed by histology and immunofluorescence at the end of the treatment cycle. H&E and Masson staining (Figure 8e) revealed that the dermal gap in the MZDH+NIR group was significantly reduced (Figure 8f), hair follicle formation was increased, collagen fiber structure was good, and collagen deposition was enhanced (Figure 8g), indicating that the tissue was in the remodeling stage. The activity of fibroblasts and angiogenesis was further evaluated through immunofluorescence staining of VEGF, α‐SMA, and CD31 (Figure 8h). VEGF is a key angiogenic factor that can be induced by HIF‐1α and promote vascular permeability, endothelial cell proliferation, and neovascularization. However, through the active release of DFO activated by NIR, the MZDH+NIR group may have already passed the peak period of angiogenesis and entered the remodeling phase earlier than other groups, consistent with accelerated transition to the remodeling phase (Figure 8i). α‐SMA is the main activated form of extracellular matrix produced by myofibroblasts, and CD31 is a marker of endothelial cell differentiation, representing vascular integrity and angiogenesis density [20]. Correspondingly, the MZDH+NIR group exhibited the strongest red fluorescence, indicating the highest expression levels of α‐SMA and CD31 in the wound skin (Figures 8j,k), confirming enhanced fibroblast activation and vigorous angiogenesis, highlighting the platform's ability to accelerate tissue regeneration and vascularization. Notably, the MZDH+NIR group consistently outperformed groups lacking either oxygenation (MDH) or DFO (MZH), supporting a synergistic model wherein localized oxygenation improvement via CAT‐like activity resolves acute hypoxia, while DFO‐mediated HIF‐1α stabilization provides sustained angiogenic signaling. As a result, the MZDH+NIR treatment demonstrates compelling capabilities to modulate inflammation, promote structured collagen deposition, and stimulate functional angiogenesis, collectively contributing to the accelerated healing of diabetic wounds.

## 3 Conclusion

In conclusion, we have developed a spray and near‐infrared activated nanozyme hydrogel to accelerate the healing of diabetic wounds by synergistically destroying the vicious cycle of oxidative stress, infection, and inflammation. The platform integrates the photothermal/electronic properties of MXene, the multienzyme activity of ZnHCF, and the iron chelate/HIF‐1 α stabilization function of DFO, and shows a strong ability in on‐demand cascade antioxidant therapy, synergistic with “3A” (antioxidant, antibacterial, and angiogenic) effect. In addition to diabetic wound management, this multifunctional and responsive nanoplatform provides a compelling strategy for treating various refractory tissue injuries, such as burns and vascular ulcers. Future exploration can focus on making the platform adapt to other treatment methods or activation mechanisms, paving the way for the next generation of intelligent wound management systems.

## 4 Experimental Section

### 4.1 Preparation of d‐Ti_3_C_2_/Zinc‐Hexacyanoferrate (MXen@ZnHCF) Heterojunctions

Add 2.0 g of Ti_3_AlC_2_ MAX particles slowly to 60 mL of HF, stirring continuously at room temperature (RT) under a N_2_ atmosphere for 44 h. To remove residual HF and other impurities, centrifuge the mixed solution and wash it with deionized water at least 7 times until the pH is 6–7. Subsequently, vacuum‐dry the precipitate after centrifugation, then add the dried powder to 50 mL of 25 wt.% TPAOH, stirring continuously at RT under a N_2_ atmosphere for 72 h. Centrifuge and wash the product with deionized water and ethanol to remove excess TPAOH, and collect all precipitates in a centrifuge tube. Then, add deionized water to the centrifuge tube and ultrasonicate the mixture for 2 h under N_2_ atmosphere and ice bath. In addition, to remove laminated MXene and other impurities, centrifuge the mixture at 3000 rpm for 15 min and collect the delaminated Ti_3_C_2_ (d‐Ti_3_C_2_).

After adding 10 mL of PVP (375 mg) solution to the d‐Ti_3_C_2_ dispersion and stirring for 5 min, add 10 mL of 22.4 mm K_3_Fe_3_(CN)_6_ solution and 10 mL of 54.1 mm ZnCl_2_ solution, respectively, and stir at RT for 30 min. Subsequently, centrifuge and wash multiple times with deionized water to obtain MXene@ZnHCF heterojunctions.

### 4.2 Preparation of MXene@ZnHCF‐DFO/HA (MZDH)

In the MXene@ZnHCF dispersion, 20 mg of EDC and 8.3 mg of NHS was added sequentially, followed by stirring at RT for 30 min. Then, 30 mg of DFO was added, and the mixture was stirred at RT for 10 h under N_2_. Subsequently, after multiple centrifugal washes, MXene@ZnHCF‐DFO was obtained. In the MXene@ZnHCF‐DFO dispersion, 10 mL of HA35000 (70 mg) was added, and the mixture was stirred at RT for 12 h under N_2_. After multiple centrifugal washes, MXene@ZnHCF‐DFO/HA was obtained. After resuspending with deionized water or physiological saline, the solution was loaded into a spray bottle to obtain the MZDH spray formulation.

### 4.3 Materials Characterization

The changes in crystal diffraction information during the MXene@ZnHCF preparation process were obtained through x‐ray diffraction (XRD, Rigaku SmartLab SE) at a scanning rate of 2° min^−1^. The surface morphology and microstructure of the samples were characterized using a field emission scanning electron microscope (SEM, ZEISS Sigma 300) equipped with an energy dispersive x‐ray spectrometer, a transmission electron microscope (TEM, FEI Talos F200X G2), and an atomic force microscope (AFM, Bruker Dimension Icon). The elemental information on the product surface was tested using x‐ray photoelectron spectroscopy (XPS, Thermo Scientific K‐Alpha) (the “Shirly” fitting boundary type and “Shirly+Linear” optimization mode was selected during data processing). Chemical structural information during the preparation process was collected by Fourier transform infrared spectroscopy (FTIR; Thermo Fisher Scientific, Nicolet iS20). The particle size distribution and surface potential of different products were tested using a nanoparticle size and zeta potential distribution analyzer (Benano90Zeta). Using the results from the UV–vis spectrophotometer (Shimadzu UV‐3600) and the Lambert‐Beer law, the absorbance of MZDH dispersion in the Vis‐NIR biological window and the fitted extinction coefficient ε(λ) at 808 nm were obtained.

### 4.4 Photothermal Performance Evaluation

The micro‐infrared thermal imaging test platform (NUTRIC 246 M) was used to monitor the temperature change of MZDH in PBS during heating under irradiation from an 808 nm laser at different powers.

### 4.5 Stability Test

The same procedure was used to determine the photothermal stability of MZDH dispersions, and the experiment was repeated 5 times. To further demonstrate the photothermal stability of the samples in a highly oxidizing environment, MDH and MZDH dispersions were prepared in systems containing varying concentrations of H_2_O_2_. Then, the temperature rise of samples containing different concentrations of H_2_O_2_ was monitored in real time for 360 s after NIR laser irradiation using an infrared thermal imager. Moreover, MDH and MZDH dispersions containing different concentrations of H_2_O_2_ were incubated at 37°C for 10 min, and the samples were analyzed by UV–vis spectrophotometry; the absorbance change at 808 nm was used as a reference for the remaining amounts.

### 4.6 Catalase (CAT)‐Like Activity Detection (Ammonium Molybdate Colorimetry)

15 mm H_2_O_2_, with a buffer system, was added to MD and MZD at different concentrations. After incubation for 5 min, the supernatant was collected after centrifugation, and 3 mg of ammonium molybdate was added. After the reaction, the sample absorbance was measured by UV–vis spectrophotometry, and the absorbance at 360 nm was used as a reference for the reaction product of ammonium molybdate and H_2_O_2_. Similarly, under the same experimental conditions, CAT‐like activities of H_2_O_2_‐MZD irradiated with different‐power lasers were measured. Under the same conditions, 50 µg/mL of MD, MZD, and MZD under 808 nm irradiation was reacted with different concentrations of H_2_O_2_ under varying conditions. Absorbance values at 355 nm were measured at different time points using a microplate reader (Synergy H1). Using a Lineweaver–Burk plot, the maximum velocity (V_max_) and Michaelis‐Menten constant (K_M_) were calculated based on the reciprocal of the reaction rate and the reciprocal of H_2_O_2_ concentration.

### 4.7 Superoxide Dismutase (SOD)‐Like Activity Detection

Add different concentrations of MD and MZD dispersions containing PBS to 30 µm riboflavin, 30 µm methionine, and 75 µm NBT. Irradiate the mixture with a 365 nm UV lamp for 5 min, centrifuge to collect the supernatant, and measure its absorbance using a UV–vis spectrophotometer. Record the absorbance at 560 nm as a reference for the blue formamide compound reduced by O_2_·^−^ from NBT. Further test the scavenging ability against ·OH. After mixing Fenton's reagent (FeCl_2_, NaHCO_3_, and H_2_O_2_), different concentrations of MD and MZD dispersions were added for reaction. Subsequently, the MB indicator was added to the supernatant after centrifugation, and its absorbance was measured using a UV–vis spectrophotometer, with absorbance at 665 nm used as a reference for the remaining ·OH. Similarly, under the same experimental conditions, SOD‐like activities of H_2_O_2_‐MZD irradiated with different‐power lasers were measured.

### 4.8 Reactive Nitrogen Species (RNS) Scavenging Detection

The antioxidant activity of MZDH in scavenging RNS was further evaluated using ABTS^+^· and DPPH· indicators. Specifically, ABTS^+^· and DPPH· solutions were incubated with MD and MZD dispersions of different concentrations for 10 min, and then the supernatant was collected after centrifugation. The absorbance was measured by a UV‐Vis spectrophotometer.

### 4.9 Biocompatibility

L929 cells were co‐incubated with nanomaterials for 24 h, with the NIR group exposed to 808 nm irradiation for 5 min. After 24 and 48 h, replace the medium with fresh DMEM and add CCK‐8 assay reagent. Return the plates to the cell incubator. After 2 h, measure the absorbance using a microplate reader.

### 4.10 Traswell Assay

L929 cells were spread at a density of 6000 cells/well in the upper chamber of a 24‐well plate, with the desired nanomaterials added. 200 µL of medium containing 1% FBS was given to the upper chamber, while 500 µL of medium containing 10% FBS was added to the lower chamber. Incubate at 37°C for 24 h. Remove the lower medium layer and fix cells with paraformaldehyde solution for 15 min. Discard the fixative and wash three times with PBS. Stain with 0.1% crystal violet solution for 30 min and observe migrating cells with Olympus IX73. Measurements were performed using ImageJ software.

### 4.11 Antibacterial Experiment

The coating plate method was employed to evaluate the bactericidal efficacy of MZDH. Firstly, 100 µL of frozen *S.aureus* was added to 50 mL of LB liquid medium for activation, then incubated on a constant‐temperature shaking incubator for 12 h (37°C, 190 rpm). Dilute the bacterial suspension in PBS, then mix 2 mL of the suspension with the nanomaterial and transfer the mixture into a 5 mL round‐bottom glass flask. Experimental groups: Control, MDH, MDH+NIR, MZDH, and MZDH+NIR. The final concentration of the materials was 50 µg/mL. Irradiation conditions: Maintain the 808 nm laser power at 1 W/cm^2^, irradiate for 5 min. All experiments were conducted in a laminar flow hood. Treated samples were placed in a bacterial incubator and incubated for 6 h. Subsequently, 5 µL was diluted in 1 mL of PBS (1x) and mixed thoroughly. 50 µL was then evenly spread onto solid agar plates. After 24 h of incubation, the number of colonies on the plates was counted.

### 4.12 Live/Dead Staining

The extent of bacterial membrane damage was further assessed by SYTO9/PI staining, and bacterial viability was determined by fluorescence intensity. All procedures and groupings were consistent. After co‐incubation, resuspend with PBS, then add 3 µL SYTO9 and 3 µL PI to each mixture. Incubate in the dark for 30 min. Then dispense 7 µL onto the center of a microscope slide and cover with a coverslip. Visualize using a confocal laser‐scanning microscope (CLSM; Zeiss LSM 980).

### 4.13 Bacterial SEM

To visually display the bactericidal effect of nanomaterials, the mixture of treated nanomaterials and bacterial suspension was transferred to EP tubes. After centrifugation, the supernatant was discarded. Each tube was added with 1.5 mL glutaraldehyde solution and fixed at 4°C for 12 h. Samples were washed three times with PBS (pH 7.4), then dehydrated with ethanol at varying concentrations. After incubation in 100% ethanol for 15 min, the ethanol was replaced with fresh ethanol. Following drying, samples were examined under a scanning electron microscope to capture bacterial morphology.

### 4.14 Intracellular ROS Clearance

Cells were cultured in a 6‐well plate for 36 h, then treated with a mixture of H_2_O_2_ and DMEM containing the desired concentration of nanomaterials. The mixture was incubated at 37°C for 40 min. Serum‐free medium was added to reduce the background. Load the DCFH‐DA probe and incubate in the dark for 25 min. Gently wash twice with pre‐warmed PBS to remove excess dye. Finally, capture images. Finally, the fluorescence intensity of intracellular ROS was observed on CLSM.

### 4.15 Western Blot

Pre‐treatment: Cell incubation and handling procedures are as described previously. Cells were cultured in 6‐well plates. Protein extraction: Add protease/phosphatase inhibitors and RIPA lysis buffer, then incubate on ice for 20 min. Transfer to the EP tube and further disrupt cells via sonication. Centrifuge at 12000 rpm for 10 min at 4°C; the supernatant constitutes the protein fraction. Quantify proteins with the BCA assay. Add 5× Loading Buffer to the remaining protein solution, then denature the proteins by heating to 95°C for 5 min. Store at −20°C; Electrophoresis: Select the concentration of the separating gel based on the size of the target protein. Ensure consistent protein loading per well. Subsequently, perform electrophoresis for protein separation following the loading gel (80 V, 15 min) and separating gel (120 V, 90 min); Blotting: Transfer proteins onto pre‐activated PVDF membranes with methanol using a “three‐layer sandwich” sponge for wet transfer, maintaining low temperature throughout; Blocking and Exposure: Block with blocking solution at 25°C for an hour. Incubate overnight at 4°C with the appropriate primary antibody in a shaking incubator. Wash with TBST buffer, then incubate with the secondary antibody. Wash the membrane again. Perform exposure with the gel‐imaging system (Tanon‐5200). Additionally, antibodies should be diluted according to the instructions. The internal control antibody was β‐actin, and the target proteins were TNF‐α, IL‐1β, IL‐6, VEGF, VEGFR2, and HIF‐α.

### 4.16 Determination of Inflammatory Factors In Vitro

Total RNA was extracted from cultured cells using TRIzol reagent (TaKaRa, Dalian, China) according to the manufacturer's instructions. RNA quality and quantity were evaluated by spectrophotometric analysis, and 1 µg of RNA from each sample was utilized for reverse transcription to synthesize cDNA. Quantitative real‐time PCR was subsequently performed using SYBR Green PCR Master Mix (Vazyme, Nanjing, China), with amplification on an ABI 7500 StepOne Plus Real‐Time PCR System (Applied Biosystems, USA). All samples were analyzed in triplicate to ensure technical consistency. β‐actin was used as an internal control to normalize expression levels, and relative gene expression was calculated using the 2^−ΔΔCt method. To confirm primer specificity and amplification reliability, a melting curve was generated at the end of each PCR run.

### 4.17 Tube Formation Experiment

Thaw the matrix adhesive at 4°C one day in advance. Pre‐cool the pipette tip and 96‐well plate to −20°C for 20 min. To ensure smooth experimental progression, all previous treatments were performed on ice. Add 50 µL of matrix gel to each well, then incubate at 37°C for 45 min. Resuspend HUVECs and plate at a density of 3 × 10^4^ cells/well. Incubate for 4 h, then observe tube formation with Olympus IX73.

### 4.18 Animal Wound Healing Experiment

Establish a diabetic model in male C57 mice. Fast mice for 12 h prior to the first modeling procedure. Administer 100 µL of STZ solution (pH = 4.5; pre‐chilled citric acid‐sodium citrate solution) intraperitoneally to each mouse in three consecutive doses, followed by one week of observation. If the blood glucose level in the tail vein is ≥10.7 mmol/L, the model is confirmed to be successful. Subsequently, anesthesia is administered, and a full‐thickness skin lesion (Φ = 8 mm) is created on the dorsal surface. The first day of treatment is designated as day 1, with a single treatment administered on day 3. Wound photographs are taken and mouse weights are recorded on days 1, 3, 5, 7, 9, 11, and 13. Groups were designated as: Control, MH, MH+NIR, MZH, MDH, MZD (dropwise), MZDH, and MZDH+NIR. On day 7, wound tissue samples were collected for H&E, Masson, and immunofluorescence staining (Day 7: IL‐6, TNF‐α, and HIF‐1α; Day 15: VEGF, α‐SMA, and CD31) to assess wound healing and tissue inflammation. Following the treatment cycle, skin tissue was similarly obtained for pathological examination. Ethical approval statement (approval no: 2023QN038): Mice were housed under pathogen‐free conditions at the Translational Medicine Research Center of Naval Military Medical University. This animal protocol was conducted ethically and approved by the Animal Care and Use Committee of the Naval Medical University.

### 4.19 In Vivo Angiogenesis

To further validate MZDH's ability to promote angiogenesis, a large‐field laser speckle blood flow imaging instrument (SIM BFI ZOOM) was utilized to track neovascularization in full‐thickness skin injuries in mice on day 7 of treatment.

### 4.20 Transcriptomics Analysis

The mice were randomly divided into three groups: the control group, MDH+NIR, and the MZDH+NIR group. On the seventh day of treatment, skin tissue samples were collected from the wound sites of the mice for sequencing and analysis.

### 4.21 Statistical Analysis

All data were presented as the mean ± SD from a minimum of three independent experiments. All data were statistically analyzed by using GraphPad Prism Software Version 9.5.0 (GraphPad Prism, USA), Origin 2021 (OriginLab Corporation, USA), and/or Image J (version 1.54a). Each experiment was performed at least three times (n ≥ 3), and the specific sample size (n) was clarified in the legends of the figures. For statistical comparison, a *t*‐test was performed to compare the data between the two groups. These results were considered significant at ^*^ *p* < 0.05, ^**^ *p* < 0.01, ^***^ *p* < 0.001, ^****^ *p* < 0.0001, and n.s. (not significant).

## Conflicts of Interest

The authors declare no conflict of interest.

## Supporting information




**Supporting File**: advs75886‐sup‐0001‐SuppMat.docx.

## Data Availability

The main data supporting the results in this study are available within the paper and its Supplementary Information. All raw and analyzed datasets generated during the study are available from the corresponding authors upon request.
