# Supplementary material for: A Sprayable Nanoplatform Breaks the Vicious Cycle of Diabetic Wounds via Photoactivated Antioxidant and Drug Delivery
Source: Adv Sci (Weinh). 2026 May 29;13(47):e75886. doi: 10.1002/advs.75886 (PMC13335989; doi:10.1002/advs.75886)
Supplement: Supplementary file 1 [file ADVS-13-e75886-s001.docx]

Supporting Information

**A Sprayable Nanoplatform Breaks the Vicious Cycle of Diabetic Wounds via Photoactivated Antioxidant and Drug Delivery**

Jiahao Guo ^1,2^, Yuanyuan Meng ^1^ Qixiang Gui ^3^, Xi-Tao Yang ^4^, Yong Fan ^2,^*, Xiaodong Zhu ^1,^*

*^1^ Department of Nanomedicine, Naval Medical University, Shanghai, 200433, P. R. China*

*^2^ Department of Chemistry, State Key Laboratory of Molecular Engineering of Polymers, Shanghai Key Laboratory of Molecular Catalysis and Innovative Materials and iChem, Fudan University, Shanghai 200433, P. R. China*

*^3^ Department of Plastic and Reconstructive Surgery of Shanghai East Hospital, School of Medicine, Tongji University, Shanghai 200120, P. R. China*

*^4^ Department of Interventional Therapy, Multidisciplinary Team of Vascular Anomalies, Shanghai Ninth People's Hospital, Shanghai Jiaotong University School of Medicine, Shanghai, 200011, P. R. China*

* Corresponding author.

*E-mail addresses*: fan_yong@fudan.edu.cn; [xdzhu26@yeah.net](mailto:xdzhu26@yeah.net)

**Experimental Section**

*Materials:* Hydrofluoric acid (HF, 48.0−51.0%), ethanol (99.7%), tetrapropylammonium hydroxide (TPAOH, 25 wt%), ammonium molybdate ((NH_4_)_2_Mo_2_O_7_, 99%), nitroblue tetrazolium (NBT, 98%), methionine (98%), riboflavin (98%), and 2, 2'-azino-bis(3-ethylbenzothiazoline-6-sulfonic acid) (ABTS^+^·, 99.5%) were purchased from J&K Scientific. 1-ethyl-3-(3-dimethylaminopropyl) carbodiimide (EDC), N-hydroxysuccinimide (NHS), methylene blue (MB, 98%), ZnCl_2_ (GR grade), FeCl_2_ (99%), NaHCO_3_ (99.5%), K_3_Fe_3_(CN)_6_ (98.5%), poly(vinylpolypyrrolidone) (PVP, M.W. 1300000), and H_2_O_2_ (30%) aqueous solution were purchased from SCR Co., Ltd. Desferrioxamine (DFO, 98%), 1,1-Diphenyl-2-picrylhydrazyl (DPPH·, 99%) was purchased from apollo scientific Ltd. Ti_3_AlC_2_ MAX (600 mesh) was purchased from 11 Technology Co.,Ltd. PBS buffer, and physiological saline were purchased from Beijing Solarbio Science & Technology Co.,Ltd. Hyaluronic acid sodium (M.W. 35000) was purchased from Shanghai Yuanye Bio-Technology Co., Ltd. Dulbecco modified eagle medium (DMEM) and fetal bovine serum were purchased from Gibco. The Cell Counting Kit-8 (CCK-8) staining kit was purchased from MedChemExpress. DCFH-DA/Rosup double staining kit, calcein acetoxymethyl ester (calcein-AM)/propidium iodide (PI) double staining kit, and Hoechst 33342 staining solution for live cells were purchased from Beyotime Biotechnology Ltd. VEGFA polyclonal antibody was purchased from Abcam Plc. VEGFR2 was purchased from Cell Signaling Technology.HIF-1α, IL-6, TNF-α, VEGF, CD31, and α-SMA antibodies were purchased from AiFang Biological. Streptozotocin (STZ, 98%), citric acid trisodium salt dihydrate (99%), and citric acid monohydrate (99%) were purchased from Yeasen Biotechnology. Purified anti-mouse CD86 and CD206 antibodies were purchased from BioLegend, Inc.

*DFO release test:* Irradiate 100 μg mL ^−1^ of MZDH dispersion with lasers of different powers. After each interval, collect the supernatant after centrifugation, test it with a UV-Vis spectrophotometer, and record the absorbance at 200 nm.

*Photothermal performance evaluation:* The micro-infrared thermal imaging test platform (NUTRIC 246M) was used to monitor the temperature change of MZDH in PBS during heating under irradiation from an 808 nm laser at different powers. Calculate the photothermal conversion efficiency (η) of MZDH according to

η = $\frac{hS(T_{Max}-T_{Surr})-Q_{Dis}}{I(1-{10}^{-A_{\lambda}})}$	(1)

where *h* is the heat transfer coefficient, *S* is the surface area of the container, *T_Max_* is the equilibrium temperature, *T_Surr_* is the surrounding temperature, *Q_Dis_* is the heat emitted by the light absorbed by the sample container, *I* represents the incident energy of laser power (mW), and A represents the absorbance at wavelength λ. Moreover, *hS* value can be calculated by:

*hS =* $\frac{mC}{\tau_{s}}$	(2)

where *m* is the mass of the sample, *C* is the heat capacity of water, and *τ_S_* is the time constant of the system.

*Hemolysis assay:* Blood was collected from the orbit of mice and stored in 1.5 mL anticoagulant EDTA tubes. The blood was centrifuged at 2000 rpm for 5 min with PBS, washed three times to collect red blood cells. Then, 0.2 mL of 4% red blood cells was compared with 0.1 mL of different concentrations of MZDH dispersion solution, and the mixture was incubated at 37 °C for 8 h. The samples were centrifuged, and the absorbance of the supernatant was measured at 540 nm using a UV-Vis spectrophotometer. The hemolysis rate was calculated using the following formula:

Hemolysis rate (%) = (sample absorption − negative control absorption) / (positive control absorption − negative control absorption) × 100%	(3)

*Scratch assay:* L929 cells were uniformly seeded in 6-well plates and cultured in DMEM medium containing 10% FBS for 24 h. A T-10 pipette tip was applied to draw a straight line perpendicular to the plate. The wells were washed 3 times with PBS to remove excess cells, ensuring a clear field of view. Resuspend the material in DMEM containing 1% FBS for medium exchange, and then incubate it with the cells. The NIR group was irradiated at 0.75 W cm⁻² for 6 min. Temperature was recorded with a handheld photothermal imager to ensure intervention remained below 45 °C. Imaging was performed at 0, 12, 36, and 72 h. The cell migration rate was calculated using the following formula:

Cell migration rate (%) = (Initial_scratch area_ - Final_scratch area_) / Initial_scratch area_ × 100%	(4)

*Cellular uptake*: L929 cells and RAW264.7 cells were seeded in confocal Petri dishes for 24 hours, and then incubated with FITC-labeled MZDH for different times. After staining with 10 μ m Hoechst 33342 for 20 min, the fluorescence imaging of cancer cells was achieved by CLSM.

**Figure S1.** (a) SEM images of Ti_3_AlC_2_ and (b) m-Ti_3_C_2_ and corresponding element mappings.

**Figure S2.** (a) An AFM image of d-Ti_3_C_2_ and (b) corresponding profile height.

**Figure S3.** High-resolution (a) Ti 2p and (b) Zn 2p XPS spectra of MXene@ZnHCF cultured in normal saline for 3 days.

**Figure S4.** Average size analysis of m-Ti_3_C_2_, d-Ti_3_C_2_, MXene@ZnHCF, MXene@ZnHCF-DFO, and MXene@ZnHCF-DFO/HA.

**Figure S5.** Infrared thermography of MZDH under 808 nm laser irradiation within 6 min.

**Figure S6.** Temperature change curve of MZDH under 808 nm laser irradiation with different power density for 6 min.

**Figure S7.** UV-Vis absorbance of MDH and MZDH incubated with different concentrations of H_2_O_2_.

**Figure S8.** Heating retention rate of different concentrations of MD and MZD.

**Figure S9.** UV-Vis absorbance of MD and MZD with different concentrations in the CAT-mimicking process.

**Figure S10.** Michaelis-Menten kinetic analysis and Lineweaver-Burk plot of (a) MD, (b) MZD and (c) MZD+NIR using H_2_O_2_ as substrate.

**Figure S11.** UV-Vis absorbance of MD and MZD with different concentrations in the ·OH scavenging process.

**Figure S12.** UV-Vis absorbance of MD and MZD with different concentrations in the SOD-mimicking process.

**Figure S13.** (a) UV-Vis absorbance of MD with different concentrations for ABTS^+^· scavenging process. (b) ABTS^+^· scavenging of different concentrations of MD and MZD.

**Figure S14.** (a) UV-Vis absorbance of MD with different concentrations for DPPH· scavenging process. (b) DPPH· scavenging of different concentrations of MD and MZD.

**Figure S15.** Nyquist curves of ZnHCF and MXene@ZnHCF under 808 nm irradiation.

**Figure S16.** Relative cell viability of L929 cells after incubation with MZDH dispersion under different laser power density for 24 h (n=5).

**Figure S17.** CLSM images of (a) L929 cells and (b) RAW264.7 cells incubated with FITC-labeled MZDH NSs for 0, 1, 2, 4, and 8 h (scale bar: 50 μm).

**Figure S18.** Quantitative analysis of the killing effect of different treatments on (c) *S. aureus* and (d) *E. coli* (n = 3). Statistical significance is assessed by unpaired Student’s two-sided t-test and asterisks indicate significant differences (***P* < 0.01, ****P* < 0.001, and *****P* < 0.0001).

**Figure S19.** Quantitative analysis of TNF-α, IL-β, and IL-6 protein expression based on Western blot results (n = 3). Statistical significance is assessed by unpaired Student’s two-sided t-test and asterisks indicate significant differences (**p* < 0.05, ***P* < 0.01, ****P* < 0.001, and *****P* < 0.0001).

The impaired healing of diabetic wounds is largely driven by a self-perpetuating cycle of inflammation-repair imbalance.^[1]^ A cornerstone of this pathology is the excessive production of ROS, coupled with hypoxia, which collectively creates a state of intense oxidative stress. This hostile microenvironment not only inflicts direct biomolecular damage but also critically dysregulates the immune response.^[2-3]^ Specifically, it drives the sustained polarization of macrophages towards a pro-inflammatory M1 phenotype, while impairing their transition to the reparative, anti-inflammatory M2 phenotype. As macrophages are central coordinators of wound healing, orchestrating granulation tissue formation, angiogenesis, and collagen deposition, this arrest in the M1 state perpetuates chronic inflammation, enhances proteolysis, and prevents the natural progression to the proliferative phase.^[4-5]^ Therefore, strategies aimed at resolving oxidative stress and facilitating the M1-to-M2 transition are considered pivotal for breaking the cycle of non-healing in diabetic wounds. Based on the three representative inflammatory factors, quantitative analysis of the TNF-α, IL-1β, and IL-6 using Western Blot (Figure S15), samples containing MZ heterojunction components exhibited a certain anti-inflammatory effect, while the expression of inflammatory factors was relatively minimal in the MZDH+NIR group, indicating that cascade antioxidant nanoplatforms exhibit significant anti-inflammatory effects.

**Figure S20.** Quantitative analysis of HIF-1α, VEGFA, and VEGFR2 protein expression based on Western blot results (n = 3). Statistical significance is assessed by unpaired Student’s two-sided t-test and asterisks indicate significant differences (**p* < 0.05, ***P* < 0.01, ****P* < 0.001, and *****P* < 0.0001).

**Figure S21.** Heat map of blood routine analysis for diabetic mice before and after modeling and treatment.

**Figure S22.** Blood biochemical analysis of diabetic mice before and after treatment, including UREA (n = 5).

**Figure S23.** Quantitative analysis of wound healing rate during treatment (n = 5). Statistical significance is assessed by unpaired Student’s two-sided t-test and asterisks indicate significant differences (**p* < 0.05, ***P* < 0.01, and ****P* < 0.001).

**Figure S24.** H&E staining of mice major organs after 28 d of different treatments.

**Figure S25.** PCA analysis of the control *vs.* MDH+NIR and MDH+NIR *vs.* MZDH+NIR groups.

**Figure S26.** The difference gene heat maps of the control vs. MDH+NIR and MDH+NIR vs. MZDH+NIR groups (p<0.05, flod change ≥ 2).

**Figure S27.** Significant enrichment of GO items for genes in the (a) control *vs.* MDH+NIR and (b) MDH+NIR *vs.* MZDH+NIR groups (Biological process: top 15; Cellular components: top 12; Molecular function: top 8).

**Figure S28.** KEGG enrichment analysis revealed downregulated genes (top 30) of the (a) control *vs.* MDH+NIR and (b) MDH+NIR *vs.* MZDH+NIR groups.

**Figure S29.** The GSEA plots of the (a) IL-17 signaling pathway, (b) chemokine signaling pathway, (c) cytokine-cytokine receptor interaction, and (d) Wnt signaling pathway obtained from regulated gene pathways using the KEGG database.

**Figure S30.** (a) H&E and Masson staining images of wound tissues on day 7 in different treatment groups and corresponding quantitative analysis of (b) dermal gap (n = 3). Statistical significance is assessed by unpaired Student’s two-sided t-test and asterisks indicate significant differences (***P* < 0.01 and ****P* < 0.001).

**Reference**

[1]	X. Jiang, J. Ma, K. Xue, J. Chen, Y. Zhang, G. Zhang, K. Wang, Z. Yao, Q. Hu, C. Lin, B. Lei, C. Mao, *ACS Nano* **2024**, 18, 4269-4286.

[2]	X. Liu, B. Chen, J. Chen, X. Wang, X. Dai, Y. Li, H. Zhou, L.-M. Wu, Z. Liu, Y. Yang, *Adv. Mater.* **2024**, 36, 2308477.

[3]	X. Liu, Z. Wan, K. Chen, Y. Yan, X. Li, Y. Wang, M. Wang, R. Zhao, J. Pei, L. Zhang, S. Sun, J. Li, X. Chen, Q. Xin, S. Zhang, S. Liu, H. Wang, C. Liu, X. Mu, X.-D. Zhang, *Nano Lett.* **2024**, 24, 4924–4935.

[4]	O. A. Peña, P. Martin, *Nat. Rev. Mol. Cell Biol.* **2024**, 25, 599–616.

[5]	J. Sun, W. Jia, H. Qi, J. Huo, X. Liao, Y. Xu, J. Wang, Z. Sun, Y. Liu, J. Liu, M. Zhen, C. Wang, C. Bai, *Adv. Mater.* **2024**, 36, 2312440.
